# Address to the Graduates in Medicine, Law, Dentistry and Pharmacy, at the Sixtieth Annual Commencement of the University of Buffalo, N. Y., June 1, 1906

**Published:** 1906-08

**Authors:** Edward N. Brush

**Affiliations:** Physician-in-Chief and Superintendent, Sheppard and Enoch Pratt Hospital, Towson, Md., Professor of Phychiatry, College of Physicians and Surgeons, Baltimore, Md.


### BUFFALO MEDICAL JOURNAL.

VOL. LXII. AUGUST, 1906. NO. 1

### ORIGINAL COMMUNICATIONS.

### Address to the Graduates in Medicine, Law, Dentistry and Pharmacy, at the Sixtieth Annual Commencement of the University of Buffalo, N. Y., June 1, 1906.

BY EDWARD N. BRUSH, M. D.

Physician-in-Chief and Superintendent, Sheppard and Enoch Pratt Hospital, Towson, Md., Professor of Psychiatry, College of Physicians and Surgeons, Baltimore, Md.

MR. VICE-CHANCELLOR, Members of the Faculties of Medicine, Law, Dentistry and Pharmacy, Members of the Graduating Classes, Ladies and Gentlemen:

I MAY not be, indeed I know that to some of you I am not, revealing a secret when I say that I am not here this morning as the one first chosen to address you. I can but feel flattered that I came second to him who was your first choice, and I most deeply regret with you, that he was prevented by the pressure of public affairs from standing in my place. I fear, moreover, that before I reach the conclusion of what I have to say my audience will wish that the choice had passed on to the next man on your list of possible speakers.

When, however, the Dean of the Medical Department of your University asked me, in behalf of himself and his fellow Deans of the departments of Law, Dentistry and Pharmacy, to address you this morning, while I knew that the preparation of an address would, in the midst of a particularly active period of professional work, be an added burden; and appreciated also my lack of ability for the task, it was not difficult to yield assent. The opportunity to return to one's Alma Mater after more than a quarter of a century's absence from her fostering care, to the city of my boyhood and young manhood, to the scenes of my professional studies and early professional work, to say some words of God-speed, and possibly some of admonition and advice, gathered from varied experiences and observations in many places, and among many men, to those just entering upon professional careers, presented attractions which it was difficult, and indeed, as you see, impossible to resist.

I am, I am told, expected to give the final address to four classes of students who have completed the prescribed course of professional study, and who today receive the diploma of this University, which marks the successful completion of their University studies, and entitles them to admission to the ranks of the profession they have chosen.

Medicine, Law, Dentistry, Pharmacy! It would be interesting, were it possible, and no doubt in some degree profitable, to know what were the incentives which led you to choose a professional career, and of more peculiar interest, could we learn, what it was that led to the particular choice which each one of you has made. We are told of a certain state in life that it "is not by any to be entered into unadvisedly or lightly; but reverently, discreetly, advisedly, soberly and in the fear of God," and in the same manner, let us hope, you have entered upon that profession which will in all probability be your life work, in which it will be for you to make or mar your career; to add to the sum of human knowledge, and happiness, to contribute to the common weal; or to pass through life, touch elbows with your fellow men and women, and leave no mark behind which shall be a record that you have lived and worked among them.

Several years ago I heard a well known college president address a class of young men about to leave their Alma Mater. His address was replete with most valuable advice and admonition delivered in an eloquent and impressive manner. One thing, which he said in closing, made a lasting impression upon me, it was something like this, I regret that I cannot quote his words *verbatim*: "Some of you will, I fear, think that opportunity awaits the man, and will wait for opportunity. Some of you will grasp the skirts of happy chance, some will breast the blows of circumstance. Some of you will make chance and circumstance subservient to your determination to do the work before you in all honesty and loyalty to yourselves and your fellowmen. Some of you will make your mark in the world, some of you will never be heard of again." What portion of this applies to the members of the classes leaving this University today? If you have entered upon your life work "discreetly, advisedly, soberly, and in the fear of God" the more cheerful part you may take to yourselves. If you have sought a professional career because you considered it a calling somewhat more dignified and genteel than trade or manufacturing, or because you thought you saw in it a way to position, influence and a comfortable means of gaining a living; if, I say, no higher motive, no love for the calling you have chosen, no desire to add by your work to your own knowledge, and the accumulating wisdom of the age, to use your calling not alone as a means to elevate yourself, but to elevate and benefit your community and your profession, then you are bound to fail, no matter how much in the eyes of the world you may seem to succeed.

You have heard much in this particular locality of a "message to Garcia," and of the man who carried it and of the lessons to be drawn from the incident. There are men and women every day, and every hour of every day, who are carrying the message delivered to them with equal zeal and faithfulness. The examples and the lessons are all about you, and he who runs may read them. Your course here has trained you to carry a message, to do a particular work, and by virtue of that training you will be expected to accomplish your mission without thought of the difficulties, without being diverted from the path by dangers, real or imaginary, or by temptations to days of pleasure and nights of ease, no matter how luring.

Training will give you the ability, zeal for your profession, the strength. It was training which sent the sailor on the Maine through dark passageways already partially flooded with water, to his Captain, to calmly salute in proper form and report "the ship is sinking, Sir!" It was training which caused the young sailor, a short time ago, on the Kearsarge to rush through the flames of burning powder and shut himself in the magazine, and save the ship—it was training which sent the nurse the other day in California across floors which were being shaken as by the hand of some mighty giant, to calmly sit down by my friend's bedside, and count his pulse. None of these men thought of self or safety; their duty, instilled by training, was the first thing which appeared before them in the hour of peril, and calmly, efficiently they went about it. Your training here has taught you how to perform your duty, how to carry your message, but constant study, accumulated experience will be necessary to permit you to carry such a message and to so deliver it, as shall lift you above the common average of men and women in your calling, and permit you to make your mark in the world.

In an article written five or six years ago, upon the opportunities of the opening century, by a United States Senator, the writer quotes from the pessimistic talk of a young college graduate as follows:

"The great principles of the law have all been announced and applied to every conceivable form of human rights and controversy. * * * In invention there may be some improvements on old and present inventions, but there will be no more Edisons, no more

Teslas. In medicine we are about on the top of the mountain. In literature the creative and fundamental things have all been done. From now on books will be mere second-hand talk. In statesmanship nothing is left except that common house-keeping which we call administrative government."

The senator goes on to say that this young man's melancholy view is that which he has found crushing the enthusiasm out of most college students he has met. If the senator himself has not formed a perverted opinion of the ideas of the college students he has met, then I believe he has been singularly unfortunate in meeting his men. The college men I have known have not as a class, nor in any large number, held such views. I know, as you know, as you have probably experienced among your associates, if not in your own career, that there comes a time to many young men when a certain pessimism seems a good thing to hold and talk.

The young graduate is sometimes led to talk of the good old days and bewail the decadence of manners and morals, and the lack of opportunity of the present. Most men get well over this by the time they have entered upon the real work of life, but some remain purblind, and go about all their days unconscious of the opportunities all about them, bemoaning the past, bewailing the present.

If there be any among you who are inclined to the belief that their lives have fallen on evil times I commend to them the wise words of a Roman philosopher who wrote in the first century of the Christian era. "We must guard" he says "against letting blame fall on our own age. This has always been the complaint of our ancestors, that manners have been corrupted, that vice reigns, that human life is deteriorating and falling into every kind of wickedness. We lament in the same strain and our descendants will do the same after us."

There may be some who rail at the times, who see no good in the present, no hope in the future, but I feel that you will agree with me in the assertion that there never has been an age in the history of the world, when, to virile and healthy manhood, life was more pure, cleanly, and hopeful than now in the Twentieth Century and here in the United States. In laboratories and in hospital wards all over the land men are seeking the cause and the prevention of disease; and seeking not for personal gain, but freely giving what they find to the common stock of human knowledge, to the general good of the race. When, in the history of the good old times, were men to be found like those soldiers of the United States Hospital Corps told of by the late Major Reed who calmly volunteered to submit to the experiment of being bitten by mosquitoes carrying the yellow fever germ. Offered extra compensation, they declined it, saying "we are not risking our lives for money; if there is anything to be gained by the experiment which shall benefit mankind and control the plague, we are willing to do our part." The experiment on these men went far toward solving the problem, but had it failed, such an exhibition of the best to be found in men is of itself a most valuable asset.

Say to scoffers and pessimists, look about you, open your eyes and unstop your ears, and above all read your histories. Cultivate your sense of proportion, the possession of which, is, after all, that which distinguishes the men of calm, strong, capable minds from those who, though vociferous and noisy, really make no impression on the age and are often a drag and hindrance. It is in fact this sense of proportion, this ability to compare experiences calmly and judiciously which is the one thing which distinguishes the sane mind from all forms of pathological mental variation. Insanity indeed, might be called in brief, a disturbance of the comparative faculty. By force of habit, in deference to custom handed down to us from those older times, the gentle sex still leaves the table when the coffee is served, but not, by any means, for the same reason as in the good old days, when the habitual drunkenness and the foul talk at the close of all formal dinners, made it necessary for them to depart. No, we are cleaner both in our lives and our speech.

In those days one-half the world knew little and cared less how the other half lived. Never in the history of time has the fortunate man paid such prompt and generous heed to the call and the needs of his unfortunate brother. What a striking and inspiring example we had of this, but a few days ago, when the cry of distress came across the continent from far away San Francisco. John Howard, a hundred years ago, was a phenomenon. His noble life and philanthropic work would, today, be matched by thousands. Corruption, you say, stalks in public places and leaves its smirch on our politics and on our financial transactions, but what of the days of Walpole or Churchill or Digby, what of the South Sea Island Bubble? "Say not thou, what is the cause that the former days are better than these? for thou dost not inquire wisely concerning this."

But let us return to the statement of the young college student, which seemed to so impress the senatorial writer I have quoted.

Has law no principles to establish? Do decision and precedent cover all matters of human controversy or human right? Have the theologians come to fixed and unchangeable opinions as to man's relations to his Maker and his responsibility both as his own and his brother's keeper? Can they always decide in questions of morals, as to the springs or sources of moral sense and steer 'twixt that which on the one hand is moral obliquity, on the other, moral defect? Has invention reached its limit, and are statesmen, having no new principles to urge, to satisfy themselves with the spoils of party victory, the gains of practical politics, and the "common house-keeping" of administrative government? Have the science and art of medicine approached the zenith of their glory? Did we accept the affirmative of this it would at once paralyze all effort deaden all ambition.

"This life were brutish did we not sometimes
Have intimation clear of wider scope
Hints of occasion infinite, to keep
The soul alert with noble discontent."

When, gentlemen of the legal profession, I approach the task of saying a few words to you, I recognise that I am treading upon unfamiliar ground, that I am about to deal with a subject as old as the human family, which has grown with the growth of the race, and has changed with the changing opinions and necessities of the times, but which today rests upon the same foundation as when Moses brought down from the mountain the ten commandments, or when Christ epitomised the whole law of his day and of all time since, or to come, in the golden rule.

That foundation is the right of the individual man, stated in our Declaration of Independence, to life, liberty and the pursuit of happiness. There may be questions and disputes as to what constitutes liberty, as to how far its enjoyment by the individual, and his pursuit of happiness, may infringe upon, or curtail, what his neighbor considers his right to the same privilege, and it is about these simple questions, I conceive, that all the disputes and contentions of the law courts, if we were able to resolve them into their fundamental elements, turn; and it is to regulate and define the relations of man to man, of community to community, of nation to nation as respects these three things, life, liberty and the pursuit of happiness, that all the laws that have been made from Moses to Solomon, from Solon to the present time have been formulated and given in one way or another the force of a "rule of action." Law has been defined as "A rule of action established by recognized authority to enforce justice and regulate duty." Naturally, as I have said, law has conformed to the changing ideas of justice and duty incident to the progress of the years and the growth of intelligence and development of civilization. No one can therefore admit, I conceive, that the application of the principles of the law has reached perfection, that nothing now remains but to conform old precedents and established decisions to new questions. Your knowledge of the history of your own profession will teach you better. The criminal law has vastly changed since men were hanged for petty theft, and equally great changes have taken place in what, I believe, are called equity proceedings, changes which I imagine would be to the men of the time of Coke and Blackstone, difficult to comprehend.

When our senatorial author was writing his essay matters were looming large on the horizon in governmental and administrative affairs which then, and still, require the most intelligent and thoughtful action of our law makers, which when brought before our courts for decision, will find few precedents in the past to guide those required to pass upon them. Our colonial possessions in the far east, and those nearer at hand, the control and regulation of large aggregations of capital, the new and complicated problems in commercial law, and in the regulation of traffic, present to you gentlemen, matters which may well engage your attention which must convince any one that matters of jurisprudence still remain which the men and courts of the past and present have not even touched upon.

Some one among you may yet do for the United States and for her new and as yet unassimilated citizens, I canot call them subjects, what Lord Cromer has done for England, and in a wider and better sense, for Egypt. From the law department of this university may yet be sent out, possibly he sits before me, some American Turgot who shall do for America what that great financial minister of Louis XVI, whose writings on finance, jurisprudence, politics in the broadest sense, taxation, philosophy and I know not what more, may well be studied today, did for France. It is of particular interest to us that this great man predicted, a quarter of a century before it took place, the inevitable separation of the American Colonies from Great Britian, if the Mother Country persisted in her course toward them, and that he pointed out to Spain the folly of the course she was pursuing toward her insular possession in the East and West Indies, a course, which doggedly followed, led, as he predicted, to their loss.

Our financiers, our doctors of the tariff, our tax regulators, have much to learn from this great man, and some of you may, by the application of his wise teaching and of the principles which have here been taught you, bring order out of what now seems to many of us almost chaos.

I have had the fortune, or misfortune, to have been in many courts, to have followed many trials and listened to many and and varied decisions. If, gentlemen, there is one thing more than another which I have carried away from those experiences, it is a sense of the dependence which you men of the law place on tradition and precedent, the difficulty you experience in getting away from them in adapting your ideas and your rulings to the changing, broader and better views of the times. Trammelled by precedent or impeded by the lack of precedent, law has seldom led in medical or sanitary reforms, and rarely followed, except at great distance and with halting steps. I say this with much deference, and with a full appreciation that I am by no means prepared to prove my assertion, but this is what has impressed me. I may be wrong, it is possible that I am not however telling you anything which you have not yourselves discovered. It has been naturally in matters medical that I have been most concerned, and upon which my observations are based.

There is one word in the language pertaining to matters with which much of my life work has been concerned, with which consciously or unconsciously you and the courts have constantly to deal. It has to do with every contract of whatever sort, with every suit at law, with every defence made for crime or misdemeanor. That word is "insanity." In the disposition of property by will, deed of trust, or other conveyance, the question is always pertinent, though happily not always of necessity raised, "is or was the person conveying this property of sound and disposing mind and memory?" The integral parts of the body politic, the families, are just as much under its dominating influence; questions of marriage, the continuation of family relations, the severance of the marriage tie, the descent of property, are all modified in more instances than is generally known by this one word "insanity." The fate of dynasties and nations has been governed by it, and pages of history stand today which would read very differently had not insanity placed its finger on some ruler or his minister and changed everything. What effect for instance had the insanity of Chatham in preventing the conciliation of the American Colonies, whose friend he was; what more serious effect the attacks of madness of George III, destined to end his days in hopeless fatuity, blind and demented.?

You will agree with me, I think, gentlemen, as to the importance of this word, referring as it does to a disturbance of those regular, orderly, methodical mental operations essential to the proper conduct of the affairs of men and nations. To you here in New York it is a word of grave import. Within your borders, supported by public taxation, involving the expenditure annually of more than $6,000,000 you have in your well equipped and excellently conducted State Hospitals in round numbers 25,000 insane. Of the more than 80,000 admitted to those hospitals since October 1, 1888, more than 50% were of foreign birth. You are somewhat unfortunately situated in that the larger proportion of immigrants land in New York City and a considerable proportion (in

1904 over 30%, or more than 260,000) remain within your borders. What proportion of these are destined to become public charges in your almshouses, prisons and State Hospitals no one can say, but the number will be sufficiently large to warrant your thoughtful attention. I am wandering a little from what I proposed to say, but in doing so I have but enlarged the scope and importance of the subject. I ventured, somewhat rashly perhaps, to criticise the application of the law to questions medical. In the famous and now historical McNaughten case, in 1843, twelve judges in England submitted answers put to them by the House of Lords on the question of responsibility, and from that day to this, with few exceptions, the knowledge of right and wrong as to the act involved, on the part of the prisoner, has been the test of responsibility for criminal or other acts. This is not the time nor is it the place to discuss the matters here involved, but I submit to you young gentlemen this broad problem of the responsibility of the insane involving many and intricate points of jurisprudence and of medico-psychology, and urge upon you the importance of its solution.

The difficulty which confronts the bench and the bar when met by this and similar problems are not far to seek. It would not be fair to ask you to be expert in medicine, whether involving questions of mental responsibility, or less important and difficult ones, but it is not, I think, asking too much of your teachers to give some instruction, or cause some to be given, to you and those who follow you, in accordance with modern ideas of mental pathology. First at Heidelberg, now in Munich, Kraepelin, the man who has done more probably than any other one man of his time to clarify and change our conception of diseases of the brain, has been giving a regular course of lectures on Forensic Psychiatry with practical demonstrations taken from the wards of his clinic, and these lectures are eagerly followed by judges and attorneys, to their profit, the benefit of their clients, and the enlightenment of the law.

You have not far to search, you see, to find matters medical which may well engage your attention, and conjoined with these and cognate to them are important subjects relating to the regulation and restriction of immigration which must engage the earnest attention of the best minds if we are to control in any measure conditions which are now injecting into the blood of the nation additional sources of pauperism, crime and disease, and imposing upon us additional burdens of taxation.

The burden of these problems is upon your shoulders, young men, and upon those of your asociates in the law. It rests there because you are supposed to be learned in the law, and able out of its provisions to apply old enactments, or devise new ones, which shall serve for their solution. You cannot, gentlemen, in your future work, like Numa, gain inspiration at the fountain of Egeria, nor may you solve your difficulties, as did Lycurgus, by consulting the Pythian oracle. You must be guided by the principles of law as announced and defined by the courts, and by the statutes enacted by various legislative bodies, under constitutional restrictions.

To the duty of each hour and each day you must bring the best that is in you of mind and heart. Honesty of purpose and act, justice in dealing with all men must, to ensure success and respect, be your guides. Before you enter upon the practice of your profession you must take a solemn oath to support and defend the Constitution of your State and of the United States. This is the highest civic duty which any man can be called upon to assume. No more important, no more sacred calling, could be yours. The higher interpretation which you put upon that call, the greater zeal which you show in answering it, the more diligence which you exhibit in perfecting yourselves for the best service to your profession and your country, the more assured will be your success, and as you succeed, as you make your mark upon the history of your times, you will reflect glory upon your Alma Mater.

What can I say, my fellow physicians, to you, and what to these others so nearly related to you in their professional work of pharmacy and dentistry, in the few moments which yet remain to me? Some of the matters which I have urged upon your fellow students of the law school apply as well to you, and you must bear your part in bringing them to a satisfactory and wise solution.

You, I am sure, have no complacent notion that medicine has about reached the mountain top. You realise, I believe, that the problems in medical science yet to be solved, notwithstanding the marvelous strides which have been made in the last quarter of a century, afford as great opportunities to the student and investigator as did those which confronted our ancestors. In anatomy there are yet unexplored fields, and this is especially true of the anatomy of the nervous system. It follows as a natural corollary that equal opportunities lie before the student of physiology. Chemistry and especially physiological chemistry affords ample scope for the most enthusiastic and energetic student, and from researches in that department we may expect, judging by the past, to achieve results which shall demonstrate the causes of many obscure diseases and add to the resources of materia-medica or enlarge and make more accurate our knowledge of the action of drugs now in use. In surgery, within the memory of men still at work in their profession, anesthesia has robbed the operating room of its terrors and wonderfully broadened surgical possibilities. Asepsis and antisepsis are of such recent application that there are many within the sound of my voice who recall the work of Pasteur and the application of his discoveries by Lister and the still more recent studies of many men in bacteriology, with the gradual development of surgical technique of the present day.

Who shall say, however, that either anesthesia or asepsis is not yet susceptible of being made more safe and easy of application on the one hand, or more certain on the other. Fellow students of medicine, for you are yet students and must so continue, in the history of the world many names connected with our profession are "writ large" on its pages. Hippocrates of Cos, whose oath of office you have taken today, Galen and Celsus, Harvey and Sydenham, Ambroisé Paré, and Louis and Laennec, Claude Bernard, Pinel, and Esquirol his pupil, Rush, the Warrens, Jenner and Lister and, in more recent times, and nearer home, to pass over a long line of men who wrought valiantly, the Flints, Dalton, Hamilton, Moore and Miner, White and Rochester here in Buffalo, accomplished for medicine and surgery things the fruits of which we are now reaping.

It is at infrequent and irregular intervals that a Jenner or a Lister, a Pasteur, a Virchow, or a Koch rises to the height of true leadership in medicine, and leaves his impress upon his times and his profession. The future holds as many opportunities for some one or more of you to reach this exalted and enviable position as it did for those who have gone before you. Tyndall tells us in one of his admirable essays, that on the Sky, that "In his efforts to cross the common bourne of the known and the unknown, the effective force of the man of science must depend to a great extent upon his acquired knowledge." Your effective force will certainly depend upon your acquired knowledge. I do not mean the knowledge you have acquired here of medical science. You have but received an introduction to the studies which must follow the reception of your diplomas. But upon the thoroughness with which you have applied your minds to your studies here, will depend your ability to continue them, away from the direction of your teachers, and upon the breadth of the general knowledge which you have brought to your aid in obtaining special knowledge, and which you must continue to cultivate and broaden, will depend in a very large measure your future success and usefulness.

The broader your culture, the more varied your intelligence, the greater number of points at which you are able to come in contact with the world of letters and science, the greater will be your advantage in the race. Cultivate then a broad general intelligence, take an interest in the affairs of your community, of your state, of the nation; above all cultivate your imagination. Don't permit it to carry you beyond the bounds of reason or into the realms of the improbable, but an imagination, capable of forming a good working hypothesis is a valuable possession to any man of science. It may, it will at times, lead him astray, but then again, it will lead him to discoveries and achievements which by the simple, cold processes of reason and of the library and laboratory would never be attained.

Tyndall in the essay to which I have referred says: "In the house of science are many mansions, occupied by tenants of diverse kinds. Some of them execute with painstaking fidelity the useful work of observation, recording from day to day the aspects of nature, or the indications of instruments devised to reveal her ways. Others there are who add to this capacity for observation a power over the language of experiment, by means of which they put questions to nature, and receive from her intelligible replies. There is, again, a third class of minds that cannot rest content with observation and experiment, whose love of causal unity tempts them perpetually to break through the limitations of the senses, and to seek beyond them the roots and reasons of the phenomena which the observer and the experimenter record.

"To such spirits, adventurous and firm, we are indebted for our deeper knowledge of the methods by which the physical universe is ordered and ruled."

The physician, of all men in the community, should be interested and interested actively in all public affairs. Who better than he, if properly trained, can advise in matters educational. You probably, here in Buffalo, have your school houses and surroundings inspected by sanitary officers; you guard the pupil against the spread of epidemic or contagious disease; you possibly have intelligent medical inspection as to the hearing and eyesight of children, so that these two important pathways for incoming stimuli to the brain may be in the best condition. You do all this possibly, and perhaps more, but what expert inquiry is made or advice given as to the capacity of the children sent to your schools to profit by all this? There are all grades of cerebral development, many varieties of cerebral receptivity or impressibility, and in many, many more instances than are recognized the backwardness and stubbornness, or moral perversity of children are due to innate brain defects of such a character as to either seriously impair or practically abolish the prospect of future mental development. Yet term after term, year after year, these pupils remain in the schools, in the same grade, to the chagrin of parent and teacher, to the detriment of fellow pupils. The hopeless experiment goes on in school after school of attempting to fit the same course of studies to all pupils, without inquiry by those best trained to judge, as to their brain capacity for that or any other course of instruction.

Not only in education, but in morals the physician, of all men, has a field for his best work. There are those who hold and teach that there is implanted in man what they term the moral instinct, or moral sense, a fundamental immutable faculty, which points out the straight and narrow path of rectitude, which applauds, or at least approves, what he does that is right; which reproves that which is wrong. Those who hold this theory seem to lose sight of the fact that the standard of morals has changed, and is changing, changing I believe for the better. Not only is this true but it differs at the present day with different nations, and among savages there is none. It cannot be denied that as civilization has advanced, as man has emerged from his primitive state, his appreciation of his relations to his fellowmen, as well as his self-appreciation, that is, his appreciation of what to himself is degrading and what is uplifting, has greatly broadened.

As Lecky says in his "History of the Rise and Influence of the Spirit of Rationalism in Europe," "Gradually, by the silent pressure of civilization a profound change passed over our public opinion. It was effected not by any increase of knowledge nor by any process of definite reasoning, but by the gradual elevation of the moral sense."

I take it that we must admit that man's moral nature results from the accumulation of certain "use habits" of the brain transmitted from generation to generation, modified by environment, and perfected, or the contrary, by education and training. In other words, heredity environment, and that includes both social and physical environment, and training, all go to make up for the individual his ability to appreciate the moral law and to respond to the requirements of the law.

One of your papers, a day or two ago, commented editorially under the caption, "Morals by Surgery," upon a case of brain surgery, the operation happily resulting in a great change for the better in the conduct and habits of the patient. Surgery unfortunately cannot attack the cases I have in mind; they result from defect rather than disease. There are numerous cases, the moral imbeciles, the so-called moral degenerates, found in our schools and colleges, and in our criminal courts, with whom the physician rather than the courts or the penal officer should deal.

If the physician is, as a citizen and a professional man, interested in those things which go for the best training and culture of the coming generation, he is all the more interested in those matters which affect the life, health, and progress of the present, for they are elements which enter into his every-day life and occupation, which must be considered at every bedside.

As man has passed across the stage of human existence from the time of the cave dweller to the present day, there have come to his nervous organization increased demands; more and more complex stimuli have demanded response from his nervous system, especially from his brain, and with the increased demand and response there has resulted a more complex and thoroughly differentiated organ. With the elaboration of structure to correspond with and meet the necessities of increased and wider function have come certain dangers or penalties. The greater the differentiation of parts, the more active the response to stimuli, the greater the instability, and consequently the more pronounced the tendency to disease.

The straining after the meretricious in everything, the desire to be seen and known of men, which seems to be the predominating social feature of the times, among many of the citizens of our country, brings as the inevitable result of the rush and push, the disappointment and ruin which so often follows from so many misguided ambitions its train of physical and mental wrecks.

An eloquent clinical teacher ("Collected works of P. M. Latham," Vol. II, p. 345), writing 70 years and more ago, said: "Among the higher and educated classes there is in this age and country (England) a wonderful striving for all the objects of wealth and honor and power. We need only think upon the strife of politics, the hazards of mercantile gambling, and the wear and tear of hard professional toil to see how many there must be who, from the common business of life, have derived both to their minds and bodies new feelings and impulses and new susceptibilities of disease. These susceptibilities belong chiefly to the nervous system."

If the rush for power, place and pelf were sufficient in its influence upon the health and mental and nervous stability of the race in the early half of the past century to attract the thoughtful consideration of medical men and call forth the warning which I have just quoted, what must be the lesson to be gained from the conditions which confront us at the opening of the present century?

I commend to you in preparing for the great life work before you, to cultivate the art as well as the science of medicine. Science is cold, observing, calculating upon phenomena and their cause. Art is live, real, warm, her ways are pleasant and her manners hopeful and cheering. I think sometimes that we forget, in our eager pursuit of exact knowledge, in our cold scrutiny of phenomena, in the self-possession to which we must school ourselves in all emergencies, that we are dealing with men and women and not mere cases, and that in our interest in the disease, we lose sight of the individual, and omit many things which would go far to his comfort and peace of mind and which may have an influence we cannot weigh or measure in the final outcome.

To your associates, the pharmacists and the dentists, what can I say? My work has been of such a character for years that I have written few prescriptions, and not having the fear of that demand, which those who seek the advice and treatment of the general practitioner make, for something to take, called medicine, I have had a lack of need of giving drugs, which has been to me gratifying, and,—shall I say it in this presence?—to my patients, of great benefit.

Drugs, medicines and pharmaceutical preparations are needed and necessary, and when needed it is fortunate that men like you are enabled by study and laboratory work and training to prepare them. We are hearing more or less in the public prints at present about Drug Trusts, proprietary preparations and the like. The presence here of a class like yours is the best assurance which the public, which takes its medicines always on trust, whether from the doctor or as advertised in the morning paper, can have of better things in store.

No one approaches a dentist without fear; he, and conscience, make cowards of us all, and I do not differ from my fellows. I have fallen into the hands of some of you, and sometimes in emergency I could not choose my doctor. When I found an American trained dentist, however, no matter where, I felt more confidence than when I approached the bungling, harsh, and not over-tidy "Zahnarzt," to whose ministrations I have had to submit on one or two occasions. Your training has been in some degree, that of the medical man; you aid and sometimes supplement his work, and it is not difficult to imagine that in time to come yours will be, as it should, regarded as a branch of the medical profession.

I commend to all of you, doctors, lawyers, dentists, pharmacists, the wise words of a citizen of this State who has made, and is destined to make, his mark upon our national history. When asked why he had chosen to assume important, trying, burdensome duties, involving public responsibility, rather than wait for a more attractive and exalted position, to which he might with much confidence aspire in the future, he said, "My feeling is that the things of the present one has the opportunity to do are substances; that the things one sees possible in the future and tries to get are shadows."

Instead of being pessimistic, in place of decrying the supposed decadence of the times, and the alleged lack of opportunity, adopt for your guiding principle the words of the great Apostle of Christianity:

"Whatsoever things are true, whatsoever things are honest, whatsoever things are pure, whatsoever things are lovely, whatsoever things are of *good* report, if there be any virtue, if there be any praise, think on *these* things."

You will find the world, if you open your eyes, full of illustrations of all of these to afford food for thought, and among your fellow men and women innumerable examples of lives based on these virtues to give you examples to imitate and emulate. Do not, I beg of you, enlist in the ranks of those who are forever crying don't, don't, but be among those who exemplify the force of that short but expressive word "do."

Citizens of Buffalo,—I had almost said fellow-citizens,—so much do I feel at home,—residents of no mean city, you have much to congratulate yourselves upon, in the beauty, wealth and influence of the place of your homes and your business. A clergyman, whose name and memory are revered in hundreds of your homes, in an address at the close of thirty-one years' pastorate of one church said:

"I remember the city of my birth and boyhood, when her jacket was homespun and her kirtle was linsey. As she grew more refined and womanly, her children clad her in silks, placed a crown on her motherly forehead and called her a queen, the Queen City of the Lakes."

The period which has elapsed since I left the halls of your University is of about the same length as that closed by this address. As I come back from time to time, your growth, your wealth, the growing beauty of your streets and residences, the activity and push of your business sections, cause me to feel in somewhat strange surroundings. In 1846 when this University was organized and the work of the department in medicine inaugurated, the population of your city was but 30,000 in round numbers. In 1855 the assessed valuation of property, real and personal, but $35,488,557.00. Today your population is more than ten times what it was in 1846 and the assessed valuation (not by any means, I take it, the real value) of your property, real and personal, together with certain franchises, which are taxed, is over 260,000,000 dollars.[1]

1. Cannot this city, upon this basis, afford for a period of, say 10 years, to add to its tax-rate, for the support of a department of letters in the University, the sum of one and one-half cents upon each one hundred dollars? This would ensure to the University an income of about $40,000.00 per annum. With this aid other contributions from private sources would inevitably be attracted and the institution be put upon a self-supporting basis. As a business venture alone, as a means of attracting money to the city, it would be a paying investment. The students now attending the school spend annually in the city for board, clothing, books, fees and incidentals, more than double this sum and the enlargement of the University would more than double it again.

Your University was chartered by the patriotic and enterprising gentlemen who were active in its inception upon broad lines, but it received practically no public aid, and has received none since. Its one department first organized, was carried on for years by the tireless, self-sacrificing devotion of the men who accepted teaching positions therein. It attracted to your city men whose fame as physicians and surgeons, teachers and authors, is world-wide. The work which these men did for medical science and for humanity cannot be estimated or measured, yet they worked so quietly, so unobtrusively, that many citizens of your town were scarcely aware that such work was being carried on in their midst. Now these other departments have been added, and you have not only a most thorough school of medicine, but of law, pharmacy and dentistry, the presence today of the members of whose faculties prevents my telling you, what you know in part, of the high estimate placed upon their teaching by professional men.

All these have been added to the resources of your city without any public aid or private subscription, except the small sum raised to erect and equip the old brown stone structure which some of you may recall on Main and Virginia Streets.

Now, and for some time, a movement has been on foot to round out, or crown, rather, the University organization by the establishment of the department or school of arts or letters,—for which the charter makes provision. Let me urge upon you men of wealth and business activities the importance, the necessity, the wisdom of bringing about this consummation. Crown your city not only as Queen of Commerce, but a Queen of Education, a leader in all that stands for the general uplift of the people of this community and, through those who shall come here for training, of still other and widely separated communities.

Fire, earthquake, the tornado may destroy your mansions, your factories and storehouses and lay waste your beautiful streets. Commercial disaster may sweep away your accumulated wealth, but neither things present nor things to come can move or destroy the value of a sound education, the opportunities for which you can here provide. The temples of Greece are in ruins, her classic groves where philosophers were wont to walk and teach are gone, but the teachings of her sages survive.

"For wisdom," we are told in Ecclesiastes, "is a defence," and the preacher goes on to say "money is a defence." He concludes, however, "but the excellence of knowledge *is* that wisdom giveth life to them that have it."

Lay the foundation of your college of Arts broad and sink them deep. Do not put your money in brick and mortar and stone, except so far as may be necessary to shelter teachers and scholars, but rather in men and books and apparatus. Attract to your school men who love art and literature and science for what they mean to them, and for what they can be made to mean to their pupils, and through them to the men and women who shall come after them. Such men are to be found, such men are eagerly longing for such an opportunity as you can give them here.

I know a few such, and I trust you are fortunate enough to know more,—men whose very acquaintance is the beginning of a liberal education. You sit by the fireside or in the woods or walk afield with them and they lead you into realms of wisdom and delight in their talks of art, literature, science; of men and affairs, of history and fiction, of moving scenes by field and flood; and the petty affairs and worries, the small or large ambitions of life are forgotten as you get a glimpse of how such a man can say:

"My mind to me a kingdom is;
Such present joys therein I find,
That it excells all other bliss
That earth affords or grows by kind."

Of the power and influence of the right kind of teachers you all know; of what they were to the school with which they were connected you can readily recall examples. Of one such man, for many years the head of Williams College, you know the estimate made by President Garfield, "a log with Mark Hopkins sitting on one end is a University."

Some months ago I received a request to state what influences in my early life had contributed, in my estimation, to whatever success has attended my professional career. While I did not think that I had accomplished enough to make an answer to the query of any value or interest, and therefore declined to answer it, my thought instinctively turned to the teachers of this University, and particularly to three with whom I was brought, as a student, and afterward, into more intimate relations. Next to what is due the dear lady, the wise and loving mother, who awakened and encouraged in me a desire for books and study, and to another, a daughter of Buffalo, whose encouragement, counsel and gentle criticism have been my chief good, I owe to the teaching and example of three men who lived and worked here in Buffalo, and who taught in this University, whatever ambition to succeed, and whatever success has come to me. Miner, White and Rochester, —men who in their day were part of the glory of your University, and who by their work and teaching and reputation were part of the glory and honor of your city.

Build whatever you erect upon the foundations which you will prepare for your department of arts and letters only after careful planning and mature deliberation. Think not of immediate satisfaction and personal gratification, but of future usefulness and stability. You will recall the laws and rules laid down by Plato for his Republic; I may be permitted to revert to some of these in urging the completion of your University.

Well-devised rules were made concerning the training of the young. The most careful attention was paid to good surroundings; nothing mean or vile was to meet the eye or strike the ear of the young scholar; music, literature and gymnastics were first taught; gentleness was to be united with manliness; beauty of form and activity of mind were to be mingled in perfect and harmonious accord. The object of education was to fit the growing child and youth to become a good citizen. The primary object was not the good of the individual as an individual, but the good of the state or the whole by making the units thereof best fitted to assume either the duties of citizens, teachers or legislators. The object of learning was the good of mankind.

Bacon somewhere says: "Men have entered into a desire of learning and knowledge, sometimes upon a natural curiosity and inquisitive appetite, sometimes to entertain their minds with variety and delight, sometimes for ornament and reputation, and sometimes to enable them to victory of wit and contradiction, and most times for lucre and profession, and seldom sincerely to give a true account of their gift of reason *for the benefit and use of man,* as if there were sought in knowledge a couch whereon to rest a searching and restless spirit, or a terrace for a wandering and variable mind to walk up and down with a fair prospect, or a tower of state for a proud mind to raise itself upon, or a fort or commanding ground for strife and contention, or a shop for profit or sale, and not a rich store-house for the glory of the Creator and the relief of man's estate."

The Athenian youth was taught that his duty and the object of his training and education were the advancement of his country's good, not his personal benefit. In his oath on being admitted to citizenship he swore to endeavor to leave his country as a result of his life, in a "better, not a worse condition."

How many of the youth of the present day or of the day when you and I, my hearers, were school boys and girls, are or were taught such high ideals by precept or example? What effect upon the growing generation does the present-day strife for power and pelf have in lowering its appreciation of the true value of learning, of the real power or influence of ideas?

Nothing mean or vile was, you will recall, to meet the eye or strike the ear of the young pupil. Are we careful in this respect as we might be? Do we appreciate and value the influence of environment in the training of our youth, as well as in the lives and conduct of our citizens in general? The growing application of good architecture, both as applied to buildings and to cities as a whole, of mural decoration, of landscape art in our parks and home surroundings, is a hopeful sign.

Not in "the magic numbers and persuasive sound" of music alone, is there power to move the living soul, but beauty keeps

"A bower quiet for us, and a sleep full of sweet dreams, and health and quiet breathing."

The opportunity is before you, men and women of Buffalo, and of Western New York. Will you seize it, will you rise to the occasion? As you do, you will be meeting a great duty as it should be met; as you neglect it, you will be hindering the birth of better things, putting off opportunities which your sons and your sons' sons should have, to attain high thinking and right living.

Much that I hope you will do, will be for posterity, and it is well that it should be. Having received much from those who have preceded you, it becomes you to hand down to those who come after you even more, for you have had greater advantages, wider opportunity, than did your ancestors. You remember the words of Ruskin (The Lamp of Memory—Seven Lamps of Architecture): "When we build let us think that we build forever. Let it not be for present delight nor for present use alone; let it be such work as our descendants will thank us for, and let us think, as we lay stone on stone, that a time is to come when those stones will be held sacred because our hands have touched them, and that men will say as they look upon the labor and wrought substances of them, 'See! this our fathers did for us."

